# Extensive drug resistant (XDR) *Acinetobacter baumannii* parappendicular-related infection in a hydrocephalus patient with ventriculoperitoneal shunt: a case report

**DOI:** 10.11604/pamj.2020.36.218.24822

**Published:** 2020-07-27

**Authors:** Cucunawangsih Cucunawangsih, Akhil Deepak Vatvani, Kalis Waren

**Affiliations:** 1 Faculty of Medicine, Universitas Pelita Harapan, Tangerang, Indonesia,; 2 Department of Microbiology, Faculty of Medicine, Universitas Pelita Harapan, Tangerang, Indonesia

**Keywords:** Neurosurgery, *Acinetobacter baumanii*, tigecycline

## Abstract

Ventricular infection due to XDR-Acinetobacter baumanii (A. baumanii) is the most severe complication after neurosurgery which associated with high morbidity and mortality. Managing A. baumanii ventriculitis/shunt infection and multiple brain abscesses is challenging since its nature that tends to be pandrug resistant to all antibiotics used. Thus, we present the first such case with problems in administration based on the available data.

## Introduction

Malfunction of ventriculo peritoneal (VP) shunt or external ventricular drainage (EVD) insertion closely related with post-neurosurgical infection which leads to longer hospitalization stay, higher costs, morbidity and mortality rate [1,2]. *A. baumannii* has become an important hospital pathogen as its ability to tolerate drying and promote resistance to various classes of antibiotics [1, 3]. This makes it challenging in treating this organism. The effective treatment of intraventricular and intravenous tigecycline and/or colistin has been reported [1, 4, 5]. In this case, we present a severe case of ventriculitis and brain abscess XDR-*A. baumannii* post tumor removal and VP shunt implantation treated without intraventricular use of antibiotic.

## Patient and observation

**Figure 1:**
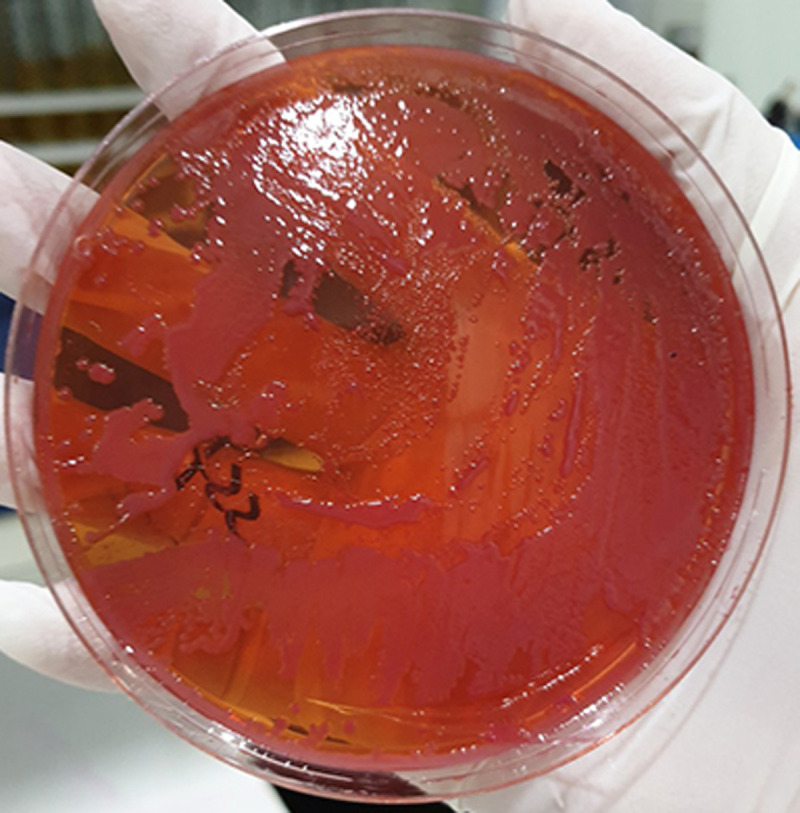
the culture appearance of *Acinetobacter baumannii* colonies on MacConkey agar

A 15-year-old female patient came to our emergency department with complains of generalized abdominal pain and tenderness in the past two days along with fever, nausea, vomiting, diarrhea and generalized weakness. The patient had a history of germinoma two years ago that was treated using open tumor removal surgery, gamma knife radiosurgery and chemotherapy. VP shunt was also placed due to obstructive hydrocephalus. The patient was then sent for whole abdominal computed tomography (CT) scan and the result was suggestive for parappendicular abscess along with free fluid in the pelvic cavity. Explorative laparotomy was then carried out which showed that there was a perforated appendix with pus around it. Post-surgery, the patient was diagnosed with sepsis. Head magnetic resonance imaging (MRI) was then carried out due to loss of consciousness and sepsis. MRI results were suggestive for ventriculitis, periventricular abscess and multiple cerebral abscesses. An EVD was carried out. Culture of cerebrospinal fluid (CSF) and peritoneal fluid on MacConkey (Figure 1) were both positive for *A. baumannii* (polymyxin B susceptibility was not tested) and were found to be resistant to 19 of the 20 drugs tested except for tigecycline (MIC = 1μg/ml) (Table 1). For this patient, meropenem 1g intravenous every 8 hours was started as empirical therapy. With this available data, a few days later antibiotic therapy was changed to intravenous tigecycline (100mg first then 50mg every 12 hours) without intraventricular administration. Repeated CSF culture still revealed XDR-*A. baumnaii* in which the patient did not show any significant clinical improvement and passed away after a while.

**Table 1:** antibiotic susceptibility of CSF and peritoneal fluid culture

Antibiotics tested	CSF		Peritoneal fluid	
	SIR^1^	MIC^2^	SIR^1^	MIC^2^
Cefazolin	R	≥ 64	R	≥ 64
Cefoperazone^*^	R	NA	R	NA
Cefotaxime^*^	R	NA	R	NA
Ceftazidime	R	≥ 64	R	≥ 64
Ceftriaxone	R	≥ 64	R	≥ 64
Cefepime	R	≥ 64	R	≥ 64
Gentamicin	R	≥ 64	R	≥ 64
Amikacin	R	≥ 64	R	≥ 64
Ampicillin	R	≥ 32	R	≥ 32
Ampicillin/sulbactam	R	≥ 32	R	≥ 32
Cefoperazone/sulbactam^*^	R	NA	R	NA
Piperacillin/tazobactam	R	≥ 128	R	≥ 128
Trimethoprim/sulfamethoxazole	R	≥ 320	R	≥320
Aztreonam	R	≥ 64	R	≥ 64
Imipenem^*^	R	NA	R	NA
Meropenem	R	≥ 64	R	≥ 16
Fosfomycin^*^	R	NA	R	NA
Tigecycline	S	1	S	1
Ciprofloxacin	R	≥ 4	R	≥ 4
Levofloxacin^*^	R	NA	R	NA

^1^SIR = Sensitive/Intermediate/Resistant; ^2^MIC = Minimum Inhibitory Concentration. MIC values expressed in μg/ml; ^*^Antibiotics susceptibility testing was done using disk diffusion method

## Discussion

*Acinetobacter baumanii* is an opportunist gram-negative bacillus that is aerobic, pleomorphic, non-motile, catalase (+) and oxidative (-). It is one of the most common pathogens associated with hospital acquired infection, which has varied clinical presentation from bacteremia to post-neurosurgical infection [2, 3, 6]. This bacterium is able to ally multiple drug-resistant genes, resulting in carbapenem-resistant *Acinetobacter baumannii* (CRAB) to pandrug resistance which have emerged become a main pathogen among critically ill or impaired immunity individual [1, 6]. Neurosurgical patients with medical devices, such as VP shunt or EVD are highly at risk getting hospital-acquired meningitis or ventriculitis [7]. There was an increased incidence of intracranial post-neurosurgical infection by *A. baumanii*, which has consequences in patient´s management due to fewer available antibiotics option [2, 6, 7]. Difficulties are strongly related to the regulation of the drugs, reporting of infections caused by these bacteria to clinical practice [4, 8]. Moreover, the increased resistance of *A. baumannii* against first-line antibiotics, which it makes challenging to cure using commercially available potent antibiotics [6, 9]. Due to the poor effectively of many intravenous antibiotics penetrate the blood-brain barrier, intrathecal polymyxin B together with intravenous β-lactam or tigecycline become a choice therapy [1, 2, 4-6]. The effectiveness of combining therapeutic strategy with intraventricular colistin has produced favorable clinical outcomes, although it has not been well studied [1, 2]. The use of antibiotic combination, whether empirical or targeted, has not been proven. Therapeutic management using antibiotics alone is usually unsuccessful; hence internal/external devices are needed for removal followed by antibiotics administration [9].

In our case, the reason for using meropenem (1), it has been stated that MDR organisms´ exhibit weakened virulence compared to other more susceptible pathogens of the same species (2), the pandrug organisms probably not indigenous patients using a long-term broad-spectrum antibiotics, whilst the definitive pathogen may not be isolated [9, 10]. High dose of meropenem have claimed successful eradication in some cases, but it was not sensitive for our patient along with a failed trial of complete meropenem therapy before starting tigecycline [7]. A case in China reported successful treated of XDR-*A. baumanii* ventriculitis using intravenous (100mg first and continued 50mg q12h) and intraventricular (2mg q12h) tigecycline with good clinical outcome [4]. Since, the use of polymixin B is inhibited commercially in our country, this led us use antibiotics systematically. Eventually, we have to use meropenem and tigecycline without intraventricular administration and our treatment did not lead to a good response. Culture of single or multiple CSF, shunt/drain and blood should be done as per-IDSA guideline for patients with ventriculoperitoneal shunt infection. Monitoring of CSF culture is also recommended to ensure that they become negative. In patients with ventriculoperitoneal shunt infections, single or multiple CSF cultures, shunt/drain and blood must be performed according to per-IDSA guidelines. Monitoring CSF cultures is also recommended to confirm they become negative [9, 10]. In our case, multiple culture, CSF and hematology parameter were done in order to monitoring the outcome of patient. However, follow up culture still revealed with XDR-*A. baumanii*. Although tigecycline is safe to use and has successful eradication in numerous data, modes of combination therapy still have better results.

## Conclusion

MDR/XDR-*A. baumnaii* is an important pathogen of hospital acquired infection with antibiotic option is limited. Therefore, management of *A. baumannii* intracranial infection is challenging for many physicians. It requires a combination of various routes and types of antibiotics in order to clear the infection.
